# Supplementary material for: ProteinShader: illustrative rendering of macromolecules
Source: BMC Struct Biol. 2009 Mar 30;9:19. doi: 10.1186/1472-6807-9-19 (PMC2672931; doi:10.1186/1472-6807-9-19)
Supplement: Additional file 1 — ProteinShader program without source code. This compressed file contains the complete ProteinShader program including associated libraries, but no source code. A README.txt file gives an overview of the ProteinShader distribution, and the index.html file in the help subdirectory has directions on getting started with the program as well as a set of tutorials. [file 1472-6807-9-19-S1.zip › ProteinShader-beta-0_9_4-binary/help/api/org/proteinshader/structure/visitor/exceptions/package-use.html]

Uses of Package org.proteinshader.structure.visitor.exceptions (ProteinShader API)


|  |  |  |
| --- | --- | --- |
| **Overview** \| **Package** \| Class \| **Use** \| **Tree** \| **Deprecated** \| **Index** \| **Help** | |  |
| PREV   NEXT | **FRAMES**    **NO FRAMES**     **All Classes** |  |


---


## **Uses of Package org.proteinshader.structure.visitor.exceptions**

| Packages that use org.proteinshader.structure.visitor.exceptions | |
| --- | --- |
| **org.proteinshader.gui** | Holds all of the Swing GUI components and their associated listeners, including class Renderer, which is registered as a listener for the GLCanvas object that is used a drawing surface. |
| **org.proteinshader.structure** | Holds the classes that store information from a Protein Data Bank file: Structure, Model, Chain, AminoAcid, Heterogen, Water, Atom, Bond, Helix, BetaStrand, Loop, *etc*. |
| **org.proteinshader.structure.io.demos** | Holds demo classes that were used for early testing of the io classes. |
| **org.proteinshader.structure.visitor** | Holds Visitor classes that know how to traverse the hierarchy of objects held by class Structure. |
| **org.proteinshader.structure.visitor.demos** | Holds demo classes that were used for early testing of the Visitor classes. |
| **org.proteinshader.structure.visitor.exceptions** | Holds exception classes needed by the Visitor classes. |

Classes in org.proteinshader.structure.visitor.exceptions used by org.proteinshader.gui

****VisitorException****
  
          Used to report that an error occurred while visiting an object.

Classes in org.proteinshader.structure.visitor.exceptions used by org.proteinshader.structure

****VisitorException****
  
          Used to report that an error occurred while visiting an object.

Classes in org.proteinshader.structure.visitor.exceptions used by org.proteinshader.structure.io.demos

****VisitorException****
  
          Used to report that an error occurred while visiting an object.

Classes in org.proteinshader.structure.visitor.exceptions used by org.proteinshader.structure.visitor

****VisitorException****
  
          Used to report that an error occurred while visiting an object.

****WriterVisitorException****
  
          Used to report that an error occurred while trying to write info on a
Visitable object.

Classes in org.proteinshader.structure.visitor.exceptions used by org.proteinshader.structure.visitor.demos

****VisitorException****
  
          Used to report that an error occurred while visiting an object.

Classes in org.proteinshader.structure.visitor.exceptions used by org.proteinshader.structure.visitor.exceptions

****VisitorException****
  
          Used to report that an error occurred while visiting an object.

---


|  |  |  |
| --- | --- | --- |
| **Overview** \| **Package** \| Class \| **Use** \| **Tree** \| **Deprecated** \| **Index** \| **Help** | |  |
| PREV   NEXT | **FRAMES**    **NO FRAMES**     **All Classes** |  |


---

# *Copyright © 2007-2008*
